# HPV-Specific Systemic Antibody Responses and Memory B Cells are Independently Maintained up to 6 Years and in a Vaccine-Specific Manner Following Immunization with Cervarix and Gardasil in Adolescent and Young Adult Women in Vaccination Programs in Italy

**DOI:** 10.3390/vaccines8010026

**Published:** 2020-01-14

**Authors:** Francesco Nicoli, Barbara Mantelli, Eleonora Gallerani, Valentina Telatin, Irene Bonazzi, Peggy Marconi, Riccardo Gavioli, Liliana Gabrielli, Tiziana Lazzarotto, Luisa Barzon, Giorgio Palù, Antonella Caputo

**Affiliations:** 1 Department of Chemical and Pharmaceutical Sciences, University of Ferrara, 44121 Ferrara, Italy; nclfnc1@unife.it (F.N.); eleonora.gallerani@unife.it (E.G.); mcy@unife.it (P.M.); gvr@unife.it (R.G.); 2 Department of Molecular Medicine, University of Padova, 35121 Padova, Italy; barbara.mantelli@unipd.it (B.M.); valentina.telatin@gmail.com (V.T.); irene.bonazzi@student.unife.it (I.B.); luisa.barzon@unipd.it (L.B.); giorgio.palu@unipd.it (G.P.); 3 Operative Unit of Clinical Microbiology, St Orsola-Malpighi University Hospital, 40138 Bologna, Italy; liliana.gabrielli@aosp.bo.it; 4 Department of Specialized, Experimental and Diagnostic Medicine, University of Bologna, 40138 Bologna, Italy; tiziana.lazzarotto@unibo.it

**Keywords:** 2vHPV vaccine, 4vHPV vaccine, IgG titers, Neutralizing antibodies, avidity index, cross-neutralizing antibodies, B-elispot, memory B cells, adolescent girls, young adult women

## Abstract

Human papillomavirus (HPV) persistent infections are associated with cervical cancer and other HPV-related diseases and tumors. Thus, the characterization of long lasting immunity to currently available HPV vaccines is important. A total of 149 female subjects vaccinated with Cervarix or Gardasil participated to the study and they were stratified according to age (10–12-year-old and 16–20-year-old). Humoral immune responses (IgG and neutralizing antibody titers, antibody avidity) and circulating memory B cells were analyzed after an average of 4–6 years from the third immunization. The humoral responses against HPV-16 and HPV-18 (and HPV-6 and HPV-11 for Gardasil) were high in both age groups and vaccines up to six years from the third dose. However, Cervarix induced significantly higher and more persistent antibody responses, while the two vaccines were rather equivalent in inducing memory B cells against HPV-16 and HPV-18. Moreover, the percentage of subjects with vaccine-specific memory B cells was even superior among Gardasil vaccinees and, conversely, Cervarix vaccinated individuals with circulating antibodies, but undetectable memory B cells were found. Finally, a higher proportion of Cervarix-vaccinated subjects displayed cross-neutralizing responses against non-vaccine types HPV-31 and HPV-45. Gardasil and Cervarix may, thus, differently affect long-lasting humoral immunity from both the quantitative and qualitative point of view.

## 1. Introduction

Human papillomavirus (HPV) infection is the most frequent sexually transmitted viral infection, which is associated with the occurrence of both benign and malignant lesions. There are more than 100 types of circulating HPVs and at least 14 are strongly associated with cervical cancer development and known as high-risk types. Most sexually active women and men acquire HPV infection during their lives and some can be infected more than once and co-infected with different types. The antibody levels that are induced by natural infection are often low and the immune responses against reinfections weak [1,2,3]. Although most HPV infections are transient and spontaneously cleared up within two years after acquisition, chronic infections occur in nearly 10% of cases with a small proportion of these infections proceeding to pre-cancerous and cancerous lesions. Persistent infection with high-risk HPV types is the fourth major cause of cervical cancer worldwide and it is also associated with ano-genital and oropharynx cancers, in both males and females, in a time frame of 15–20 years after acquisition or even less (5–10 years) in persons with a weakened immune system. Oncogenic HPV-16 and HPV-18 are known to cause at least 70% of cervical cancers, whereas other high-risk types, such as HPV-31, 33, 39, 45, 51, 52, 56, 58, 59, and 68, cause a further 20% [4,5,6].

A vaccine that induces long-term immune responses and protection against oncogenic HPV types is therefore of outmost importance in preventing cervical cancer and other HPV-related diseases and tumours. Prophylactic HPV vaccines in widespread use since 2006/2007 [7,8] include the AS04-adjuvanted bivalent vaccine (2vHPV; Cervarix, GSK, Verona, Italy) and the aluminium hydroxyphosphate sulfate salt-adjuvanted quadrivalent vaccine (4vHPV; Gardasil, Merck, Rome, Italy) [9], which also exhibit some degree of cross-protection against non-vaccine high-risk HPV types 31, 33, and 45 [10,11,12]. Further, in 2014, a nonavalent vaccine (9vHPV; Gardasil 9, Merck) has been licensed by the FDA and then approved in several countries [13]. Although manufactured by different methods, in insects (Cervarix) and yeasts (Gardasil), all of the vaccines are formulated as virus-like particles (VLPs) of recombinant L1 capsid proteins of oncogenic HPV-16 and HPV-18. However, the Gardasil vaccine also targets low-risk HPV-6 and HPV-11 that are responsible for 90% of genital warts and laryngeal papillomas and the Gardasil 9 vaccine includes the VLPs of other five oncogenic types (31, 33, 45, 52, 58). The main target of vaccination are young girls from 9 to 12 years of age, before they become sexually active and exposed to the virus, although concurrent implementation programs targeting older-ages broaden the coverage, as major risk for HPV infection is in the years after sexual debut [14,15]. More recently, some countries have also started to vaccinate boys, since vaccination prevents genital cancers and warts in both males and females [16,17]. Immune correlates of protection are not entirely clear yet, however experimental evidence indicate neutralizing antibodies (nAbs) as the main mechanism of protection. All of the vaccines indeed elicit high titres of potent, type-specific nAbs that prevent infection by transudation or exudation through the epithelium at the site of infection [8,18,19] and induce protection against cervical cancer that is associated to the vaccine HPV with approximately 100% efficacy [20,21,22,23]. Indeed, follow up studies report long-term immune responses and protection up to 10–12 years for Cervarix and Gardasil [24,25,26] and up to five years for Gardasil 9 [13,27]. However, continued monitoring is important in identifying potential signals of waning, especially in women that are vaccinated after adolescence.

Here, we have compared the long-term immunogenicity of Cervarix and Gardasil vaccines in two cohorts of preteen/adolescent girls (aged 10–12 years) and young adult women (aged 16–20 years), vaccinated in the context of current vaccination and implementation programs (targeting older-ages), to assess whether, and to what extent, the type of vaccine might impact the quality, quantity, and maintenance of these responses in two age groups. Different types of humoral immune responses (IgG and nAbs titers, antibody avidity, and memory B cell responses) were analysed after an average of 4–6 years from the third immunization. Indeed, experience with other vaccines indicate that both circulating antibodies and memory B cells may mediate protection and, in some cases, vaccine efficacy has been observed also in the absence of circulating antibodies [28]. As the role of memory B cells is not completely clear in the case of HPV vaccination, we measured their persistence in comparison to antibody levels and properties.

## 2. Materials and Methods

### 2.1. Study Design

**Figure 1:**
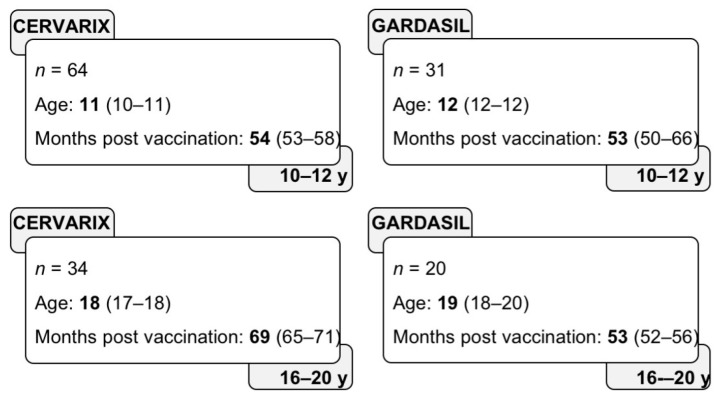
Study groups. For each age and vaccine group, the number of subjects, their median age (with interquartile range), and the time of sample collection after the third immunization (presented as median and interquartile range) are shown.

The aim of this investigator-initiated observational study was the evaluation and comparison of memory immune responses of Cervarix and Gardasil vaccines after an average of 4–6 years from the third vaccine boost in the target adolescent population of organized vaccination programs (10–12-year-old, 10–12 y) and in young adults (16–20-year-old, 16–20 y) in Italy (Figure 1). In addition, 10–12 year-old girls (*n* = 53) that were vaccinated with Cervarix were enrolled at months 1–6 after the third immunization. Enrolment was carried out at the public health districts of Padova (Veneto region) and Bologna (Emilia-Romagna region), where Gardasil and Cervarix, respectively, were offered by organized vaccination (9–12-year-old) and implementation (older ages) programs. In each district, girls who had previously completed the vaccination schedule were invited by letter to participate in the study, which consisted in blood sampling, to measure the persistence of immune response elicited by vaccination. The exclusion criteria were fever and other symptoms of active infection in the three weeks before blood collection, presence of chronic inflammatory diseases, immunosuppressive therapy, and pregnancy. Unvaccinated, aged-matched healthy donors (*n* = 24) were also enrolled as controls to establish the cut-off of the immunological assays. The Ethics Committees of Padova University-Hospital and Bologna St Orsola-Malpighi University Hospital (Protocol no 2413P) approved the study and conducted according to the principles expressed in the Declaration of Helsinki.

### 2.2. Samples Purification

Whole peripheral blood was collected in spray-coated sodium citrate tubes for the purification of plasma and peripheral blood mononuclear cells (PBMCs). Briefly, whole blood was centrifuged at 2500 rpm (Megafuge 1.0R, Heraeus) for 7 min. to collect plasma, whereas the cellular fraction was loaded on Ficoll density gradient (GE Healthcare) to purify PBMCs, as previously described [29,30]. Plasma was stored at −20 °C and purified PBMCs were suspended in 90% foetal bovine serum (FBS) (Lonza) and 10% DMSO (Sigma-Aldrich) and then preserved in liquid nitrogen until use. A small aliquot of blood was also collected to obtain sera and then stored at −20 °C until use.

### 2.3. Plasmids

Dr. John T. Schiller kindly provided plasmids p6sheLLr+, p16sheLL, and p18sheLL, containing L1/L2 genes of HPV6, 16 and 18 (Laboratory of Cellular Oncology, NIH, Bethesda), whereas Dr. Martin Müller kindly provided plasmid p11L1h containing the L1 gene of HPV11 (German Cancer Research Center, Heidelberg). Plasmid DNAs were amplified in XL-Gold ultracompetent cells (Stratagene) and purified by means of a commercial kit (Plasmid Maxi kit, Qiagene, Milan, Italy).

### 2.4. VLPs Preparation

The preparation and purification of HPV type-specific virus like particles (VLPs) was undertaken according to the protocol of Buck et al. [31]. The 293TT cell line was kindly provided by John T. Schiller (NIH, Bethesda) and then grown in Dulbecco’s modified Eagle’s Medium (DMEM) (Gibco Life Technologies) containing 10% FBS, 1% antibiotic-antimicotic solution, 1% glutamax, 1% non-essential aminoacids (all from Gibco Life Technologies), and 250 μg/mL hygromycin B (Sigma-Aldrich). For each HPV type-specific VLP, 293TT cells (7 × 10^6^) were seeded in 75 cm^2^ flasks and incubated 16–18 h at 37 °C up to 50% confluence. Cells were lipofected (Lipofectamin 2000, Invitrogen) with 20 μg of plasmid DNA, according to manufacturer’s instructions, which were incubated at 37 °C and then collected 48 h later. The cells were washed in Dulbecco’s phosphate buffered saline (D-PBS) containing 9.5 mM MgCl_2_, transferred to new eppendorf tubes and centrifuged at 2000 rpm (Centrifuge 5418R, Eppendorf) for 7 min. The cellular pellet was resuspended in D-PBS (1.5× the cellular volume) and then lysed with Triton X-100 (1/20 of the volume), 0.1% Benzonase (Sigma–Aldrich, Milan, Italy) 0.1% Plasmid Safe (Epicentre, Madison, WI, USA), and 1 M ammonium sulphate (pH 9) (1/40 of the volume) at 37 °C for 24 h. The cell lysate was added with 0.17 M NaCl, incubated on ice for 20 min. and centrifuged at 10,000 rpm at 4 °C. The supernatant was left on ice for two hours and VLPs were purified by means of an iodixanol gradient (Sigma–Aldrich) at 50,000 rpm for 3.5 h (rotor SW55ti, Beckman Coulter, Milan, Italy). Fractions were collected from the bottom of the tube, read for protein concentration (BCA protein assay kit, Pierce, Milan, Italy), and stored at −80 °C until use. The fractions were analyzed by SDS-PAGE, silver staining (Silver stain kit, Pierce) and western blot using mouse anti-L1 primary IgG antibodies (HPV6/11: Antibodies-online; HPV16: Santa Cruz Biotechnology, Dallas, Texas; HPV18: Abcam, Cambridge, UK), and an anti-mouse HRP-secondary antibody (Abcam). For each type-specific VLP, all of the fractions positive for L1 derived from several transfections and purifications procedures were pooled together to obtain a single type-specific VLP stock to be used for all immunological assays (IgG Elisa, avidity index, B-cell Elispot). The protein concentration of each VLP stock was measured by BCA and disposable aliquots (25 μg) were stored at −80 °C until use. All of the plasticware used in these procedures was siliconized (StarLab, Milan, Italy).

### 2.5. Pseudovirion-Based Neutralization Assay (PBNA)

HPV type-specific pseudovirions (PsVs) encapsidating a SEAP reporter plasmid, their preparation and purification, and the neutralization assays on serum samples were done as described previously [32]. Briefly, pseudovirions were obtained by the transfection of 293TT cells with equal amounts of p16shell, p18shell, p31shell, or p45shell plasmids and the reporter pYSEAP plasmid. For the PBNA, 293TT cells were seeded in 96-well plates at a concentration of 30,000/well in neutralization buffer (DMEM without phenol red, 10% of FBS, 1% of Glutamax™, 1% of non-essential amino-acids) grown for 2–5 h at 37 °C and incubated with HPV PsVs alone or previously mixed with sera (two-fold dilution from 1:40 to 1:163,840) in duplicate wells. After 72 h, cell supernatants were tested by using the SEAP Reporter Gene Assay [32]. The neutralization titer was determined as the reciprocal of the final dilution of serum that yielded <50% of mean RLU measured with PsVs alone and reported as ED50 (effective dose producing 50% response). The limit of quantification of the PBNA was set at 40 ED50. The serum samples with neutralization titers equal or higher than 40 ED50 were considered to be positive. Serum samples with neutralization titers less than 40 ED50 were assigned a value of 1 for the purpose of geometric mean titer (GMT) calculation.

### 2.6. Determination of IgG Titers and Avidity

HPV type-specific IgG titers were measured by ELISA in 96-well plates (half area 96-well plate high-bind, Fischer Scientific) that were pre-coated with 100 μL/well of each HPV type-specific VLP (2 mg/mL in PBS) at 4 °C for 18–20 h. The plates were washed with 0.05% Tween in PBS, incubated with 1% bovine serum albumin (BSA, Sigma-Aldrich) in PBS for one hour and then with plasma samples (50 μL) for two hours at 37 °C. The samples were serially diluted in PBS containing 1% BSA and then tested in duplicated wells. After extensive washing with 0.05% Tween in PBS, the immunocomplexes were detected by incubation with a goat anti-human IgG-HRP (Abcam) for one hour and the addition of ABTS substrate (Thermo Scientific, Milan, Italy) for 30 min. at 37 °C [33]. The optical density at 620 nm was determined with “Sunrise optical reader” (Tecan) and then analyzed as previously described [34,35]. For each type-specific VLP, the cut-off values were determined by analyzing the plasma of 24 unvaccinated healthy donors at 1:100 dilution as the mean OD (±3 SD): 0.374 for HPV-16; 0.346 for HPV-18; 0.388 for HPV-6; and, 0.346 for HPV-11. In each plate, blank (wells with PBS-1% BSA alone), negative controls (wells with a pool of plasma of unvaccinated subjects), and positive controls (wells with a pool of plasma of vaccinated subjects) were included.

The avidity of antigen-specific IgG was evaluated on selected samples with high IgG titers by a modified ELISA [36,37]. Briefly, 96-well plates were coated with each HPV type-specific VLP, washed, and then blocked with PBS-1% BSA, as described above. Based on the ELISA IgG titers, selected plasma samples were diluted in PBS-1% BSA to obtain an OD of 1 ± 0.5, added (50 μL) to the wells for two hours at 37 °C and washed as above. Negative and positive controls were included in each plate. The plates were then incubated with 50 μL/well (duplicate wells) of 0.5 to 5 M GuHCl for each sample (that removes the low avidity antibodies while high avidity antibodies remain bound) for 15 min. at room temperature under gentle agitation. Untreated samples and control wells were instead incubated with PBS alone. After extensive washing, immune complexes were detected, as described above. For each sample, the mean OD of duplicates were calculated and percentage of binding was determined as ((treated OD/untreated OD) × 100). The avidity index (AI) is the extrapolated molar concentration (M) of GuHCl required to reduce the absorbance of the untreated, control well by 50%.

### 2.7. Polyclonal Stimulation of Memory B-Cells and B-Cell Elispot

B-cell Elispot was standardized for several parameters, including the size of well-plates (24 vs 96) for cell stimulation, the concentration of stimuli, the incubation time (5 vs 6 days) (Supplementary Material Figure S1A), and the number of cells/well (Figure S1B). Based on these preliminary tests, frozen/thawed PBMCs were diluted in RPMI 1640 medium containing 10% FBS (0.5 × 10^6^/mL), seeded in 24-well plates (1 mL/well, duplicate wells), and grown unstimulated or stimulated with ODN (3 μg/mL), *S. aureus* Cowan I (1:10.000), and Pokeweed (0.1 μg/mL) at 37 °C for five days. The cells were then washed three times, counted by the Trypan blue exclusion method, resuspended in culture medium (1.25 × 10^6^/mL for antigen-specific B-cell Elispot; 6.25 × 10^4^/mL for total IgG B-cell Elispot) and added to 96-well Elispot plates. B-cell Elispot assays were performed by means of a commercial “Human IgG B-cell Elispot” kit (Mabtech, Nacka Strand, Sweden), according to the manufacturer’s instructions with some modifications. Briefly, 96-well plates (Maipswu, Millipore, Milan, Italy) were pre-washed with 70% ethanol for two minutes and water, and then pre-adsorbed, at 4 °C for 20–24 h, with 100 μL of an anti-human IgG capture antibody (MT91/145; 15 μg/mL) to quantify the total number of B cells secreting IgG, or with 100 μL of each type-specific VLP (12.5 μg/mL) to quantify the number of HPV type-specific B cells. After extensive washing with PBS, 200 μL of unstimulated and stimulated PBMCs (1.25 × 10^6^/mL) were seeded in each HPV type-specific plate (duplicate wells), whereas 200, 100, and 50 μL of unstimulated and stimulated PBMCs (6.25 × 10^4^/mL) were added (duplicate wells) to the total IgG B-cell Elispot wells and, when required, volume adjusted up to 200 μL with medium. In all plates, blank wells containing 200 μL of culture medium alone were included. After incubation at 37 °C for two hours, the plates were washed and processed with an anti-human-IgG-biotin detection antibody, according to the kit’s instructions. The spots were counted while using an Elispot reader (AElvis, Hannover, Germany). The percentage of each HPV type-specific memory B cells was calculated as [antigen-specific B cells (SFC/10^6^ PBMC)/total memory B cells (SFC/10^6^ PBMC)] × 100.

### 2.8. Statistical Analysis

The Mann–Whitney test was used to compare the difference between two independent groups, while the correlations were analyzed by Spearman’s rank test. Differences in the frequencies were analyzed by the Fisher’s exact probability test. P values of less than 0.05 were considered to be statistically significant. Statistical analysis was performed while using the softwares R [38] and Rstudio [39] with the packages Tidyverse [40] and Scales [41].

## 3. Results

### 3.1. Study Subjects

A total of 149 female subjects that were vaccinated with Cervarix (*n* = 98) (at month 0, 1, 6) or Gardasil (*n* = 51) (at month 0, 2, 6) participated to the study and were stratified according to age. The Cervarix and Gardasil cohorts included 64 and 31 subjects, respectively, in the 10–12 y group, and 34 and 20 subjects, respectively, in the 16–20 y group. All of the participants were enrolled after the third vaccine immunization (average 4–6 years) (Figure 1).

### 3.2. Long-Term Higher Antibody Levels in Cervarix-Vaccinated Women

**Figure 2:**
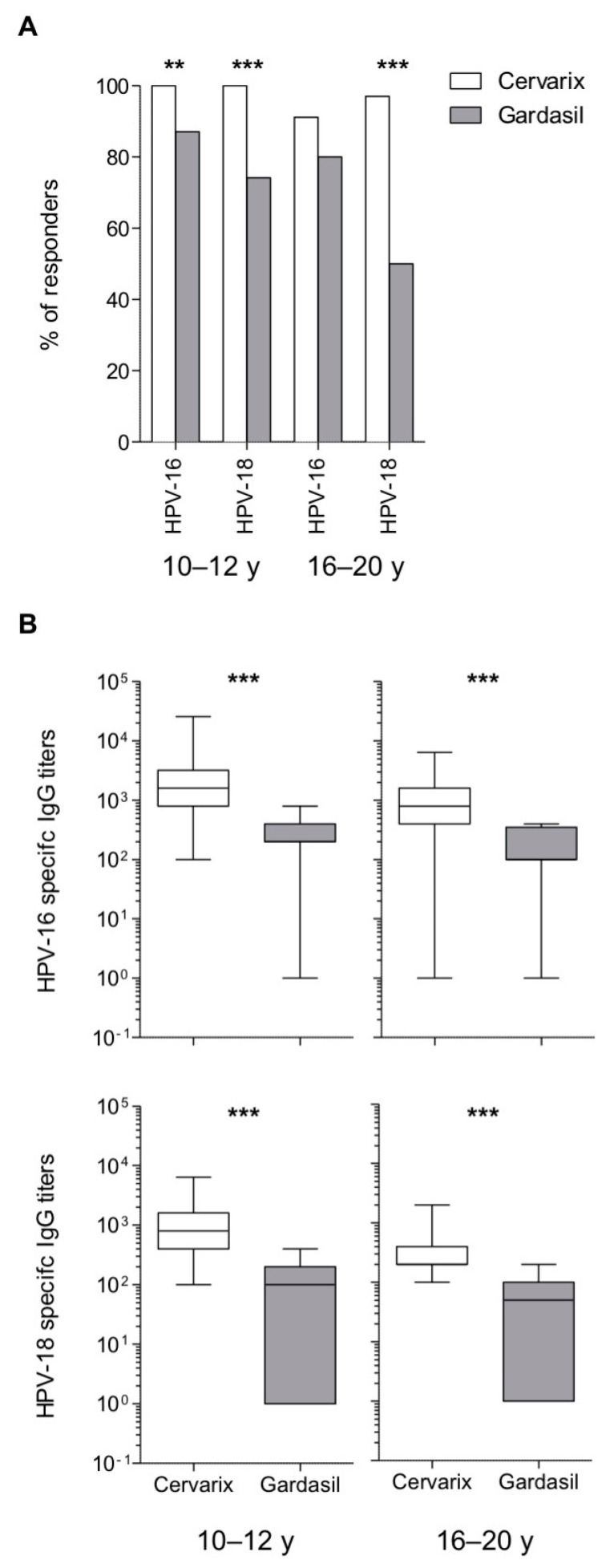
Maintenance of HPV-16 and HPV-18 specific IgG. (**A**) Frequency of study subjects with detectable binding HPV-16 and HPV-18 specific IgG. (**B**) HPV-16 and HPV-18 specific IgG titers measured by ELISA shown as box and whiskers plots. Statistical comparisons were made using Fisher’s exact probability test (**A**) and Mann-Whitney test (**B**), ** *P* < 0.01, *** *P* < 0.001; for HPV-16 10–12 y, *n* = 64 (Cervarix) and *n* = 31 (Gardasil); for HPV-16 16–20 y, *n* = 34 (Cervarix) and *n* = 20 (Gardasil); for HPV-18 10–12 y, *n* = 60 (Cervarix) and *n* = 31 (Gardasil); for HPV-18 16–20 y, *n* = 33 (Cervarix), and *n* = 20 (Gardasil).

The IgG seropositivity rates for HPV-16 and HPV-18 were significantly higher (91–100%) in both Cervarix age groups as compared to the corresponding Gardasil cohorts (50–87%) after 4–6 years from the third boost vaccination (Figure 2A), although the results in the 16–20 y group were not significant for HPV-16. Hence, Cervarix ensures the long-term duration of humoral responses against both HPV-16 and HPV-18 in all adolescents (10–12 y individuals) and in more than 90% of young adults (16–20 y individuals), whereas the percentages of subjects with detectable IgG titers is lower in Gardasil-vaccinated women, particularly in young adults for HPV-18 specific-IgG.

Similarly, HPV-16 and HPV-18 specific IgG titers were significantly higher in Cervarix-vaccinated women for both age groups (mean IgG titers in 10–12 y: HPV-16=2352, HPV-18=1125; mean IgG titers in 16–20 y: HPV-16 = 1429, HPV-18 = 439) as compared to Gardasil (mean IgG titers in 10–12 y: HPV-16 = 255, HPV-18 = 119; mean IgG titers in-16–20 y: HPV-16 = 165, HPV-18 = 70) (Figure 2B).

**Figure 3:**
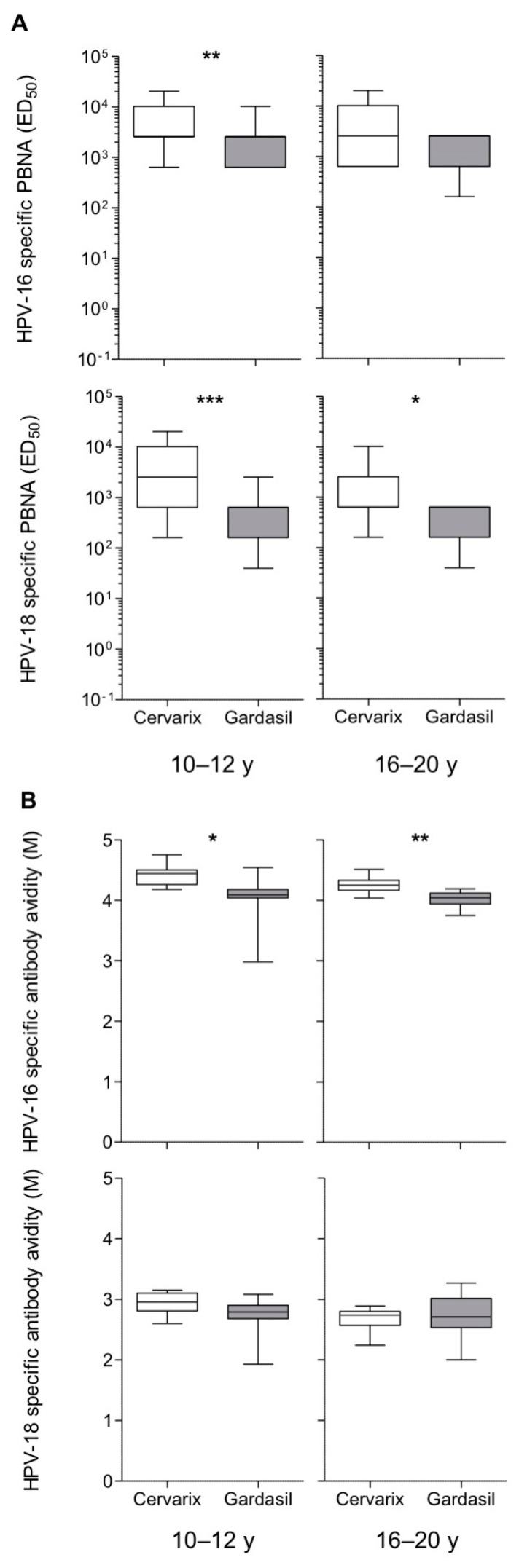
HPV-16 and HPV-18 specific nAbs and IgG avidity. (**A**) HPV-16 and HPV-18 specific nAbs measured by the PBNA, in ELISA responders, reported as ED_50_ and shown as box and whiskers plots; for HPV-16 10–12 y, *n* = 62 (Cervarix) and *n* = 27 (Gardasil); for HPV-16 16–20 y, *n* = 31 (Cervarix) and *n* = 16 (Gardasil); for HPV-18 10–12 y, *n* = 58 (Cervarix) and *n* = 23 (Gardasil); for HPV-18 16–20 y, *n* = 32 (Cervarix) and *n* = 10 (Gardasil). (**B**) Avidity of HPV-16 and HPV-18 specific IgG, measured in a subgroup of ELISA responders, reported as molarity (M) and shown as box and whiskers plots; for HPV-16 10–12 y, *n* = 10 (Cervarix) and *n* = 7 (Gardasil); for HPV-16 16–20 y, *n* = 10 (Cervarix) and *n* = 8 (Gardasil); for HPV-18 10–12 y, *n* = 10 (Cervarix); and, *n* = 7 (Gardasil); for HPV-18 16–20 y, *n* = 10 (Cervarix) and *n* = 8 (Gardasil). Statistical comparisons were made while using Mann–Whitney test, * *P* < 0.05, ** *P* < 0.01, *** *P* < 0.001.

In line with these results, the titers of nAbs after 4–6 years from vaccination were significantly higher in Cervarix-vaccinated women of both age groups and HPV subtypes (values that range from 1480 to 6613 in the Cervarix-vaccinated women and from 436 to 2133 in the Gardasil-vaccinated women), even though the difference was again not statistically significant for HPV-16 in the older age group (Figure 3A). We next evaluated the presence and titers of cross-nAbs against HPV-31 and HPV-45. Gardasil-vaccinated subjects did not show nAb against HPV-45, and only a small proportion (17%) of those aged 10–12 y had HPV-31 cross-nAbs (Supplementary Material Figure S2, titer range: 1–160). Conversely, 53 and 33% of Cervarix-vaccinated subjects aged 10–12 y and 16–20 y, respectively (Supplementary Material Figure S2), had HPV-31 cross-nAbs (titer range: 1–2560 for 10–12 y and 1–640 for 16–20 y). In addition, 23 and 3% of Cervarix-vaccinated subjects aged 10–12 y and 16–20 y (Supplementary Material Figure S2), respectively, had HPV-45 cross-nAbs (titer range: 1–160 for 10–12 y and 1–40 for 16–20 y).

Finally, the IgG avidity index was determined in a small subset of vaccinees and resulted slightly, but significantly, higher for anti-HPV-16 antibodies in Cervarix-vaccinated women of both age groups (4.4 and 4.2 for 10–12 y and 16–20 y groups, respectively) than the corresponding Gardasil cohorts (four for both age groups). Instead, for anti-HPV-18 antibodies the avidity index was, in general, lower (ranging from 2.7 to 2.9) than for anti-HPV-16, whereas it was comparable among Cervarix and Gardasil vaccinees (Figure 3B).

Overall, these results indicate that Cervarix induces long-lasting humoral immunity in both adolescents and young adult women against HPV-16 and HPV-18 subtypes. In contrast, after Gardasil immunization, measurable antibodies against both subtypes are present in a lower number of vaccinated subjects and at lower levels.

### 3.3. Maintenance of Long-Term Memory B Cells in Both Cervarix- and Gardasil-Vaccinated Women

**Figure 4:**
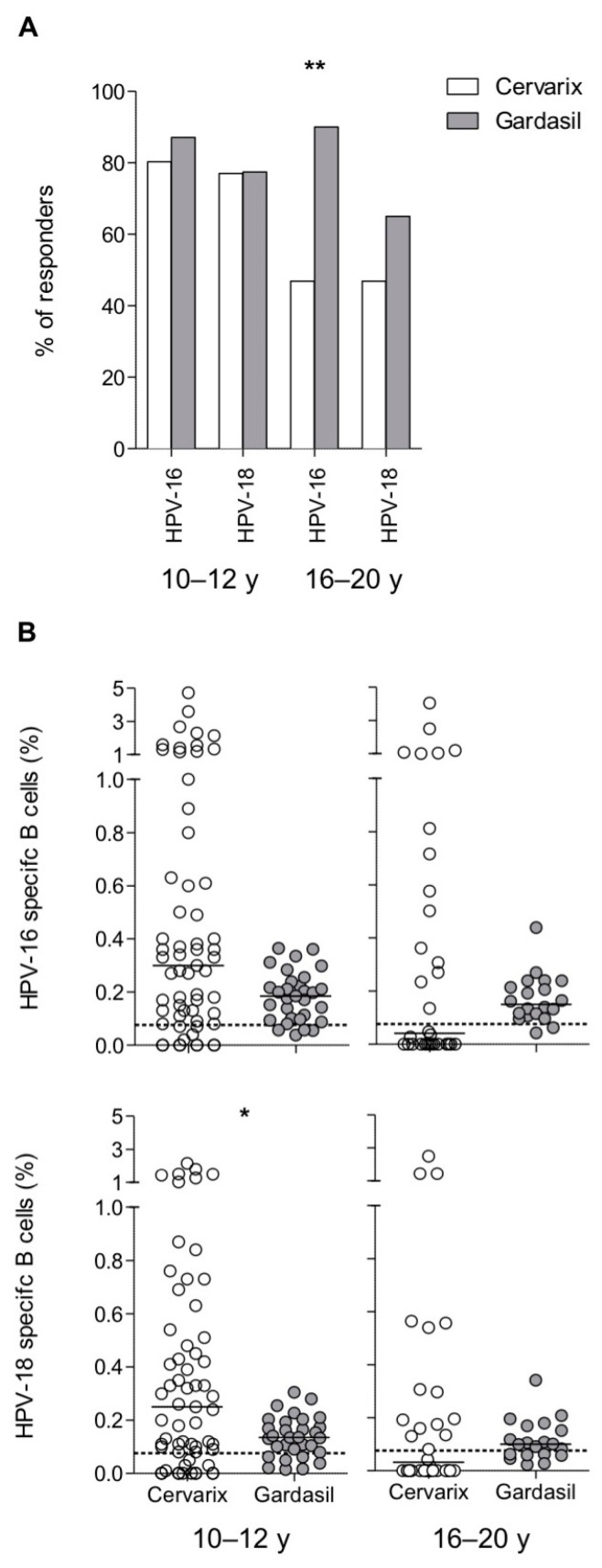
Maintenance of HPV-16 and HPV-18 specific memory B cells. (**A**) Frequency of study subjects with detectable HPV-16 and HPV-18 specific memory B cells. (**B**) HPV-16 and HPV-18 specific memory B cells measured by B cell Elispot assay. Each dot represents a single donor, solid lines are the medians and dashed lines are the cut-off values to determine the B cell Elispot positivity. Statistical comparisons were made using Fisher’s exact probability test (**A**) and Mann–Whitney test (**B**), * *P* < 0.05, ** *P* < 0.01; for HPV-16 10–12 y, *n* = 61 (Cervarix) and *n* = 31 (Gardasil); for HPV-16 16–20 y, *n* = 32 (Cervarix) and *n* = 20 (Gardasil); for HPV-18 10–12 y, *n* = 61 (Cervarix) and *n* = 31 (Gardasil); for HPV-18 16–20 y, *n* = 32 (Cervarix) and *n* = 20 (Gardasil).

We next sought to analyze the persistence of vaccine-induced memory B cells by means of B cell Elispot. Detectable HPV-16 and HPV-18 specific memory B cells were observed in a similar proportion after both Cervarix and Gardasil vaccination in the 10–12 y groups (Figure 4A). Conversely, and in contrast to serological measures, a higher percentage of Gardasil-vaccinated young women maintained detectable memory B cells after vaccination, especially against HPV-16 (47% after Cervarix and 90% after Gardasil vaccination, Figure 4A). Hence, Gardasil was more effective than Cervarix in the 16–20 y groups, while both vaccines ensure long-term memory B cells in more than 75% of vaccinated adolescents.

Despite this, Cervarix induced higher numbers of HPV-specific memory B cells, especially in the younger age group with statistical significance only for HPV-18 (Figure 4B).

Together, these data indicate that both vaccines induced long-lasting memory B cells in the majority of adolescents, although those that were vaccinated with Cervarix presented a higher number of HPV-18-specific B cells. Conversely, HPV-16-specific B cells were maintained in a larger proportion of Gardasil-vaccinated young adults.

### 3.4. Maintenance of HPV-6 and HPV-11-Specific Responses After Vaccination with Gardasil

While Cervarix only contains antigens from two HPV strains (HPV-16 and HPV-18), Gardasil also immunizes against HPV-6 and HPV-11 subtypes. HPV-6 specific IgG were maintained in 74% of 10–12 y and in 55% of 16–20 y vaccinated individuals, while the proportion for HPV-11 responders were higher (94% and 70%, respectively, Supplementary Material Figure S3A). Notably, IgG titers against both subtypes and in both age groups ranged from 105 to 406, thus in a range similar to HPV-16 and HPV-18-specific, Gardasil-induced, IgG (compared Figure 2B with Supplementary Material Figure S3B). In addition, more than 85% individuals maintained HPV-6 and HPV-11 specific memory B cells (Supplementary Material Figure S3C,D). These results confirm that Gardasil induces long-lasting memory B cells against all four HPV types, irrespective of the age of vaccination.

### 3.5. Antibodies and Memory B Cells are Independently Maintained After Vaccination

**Figure 5:**
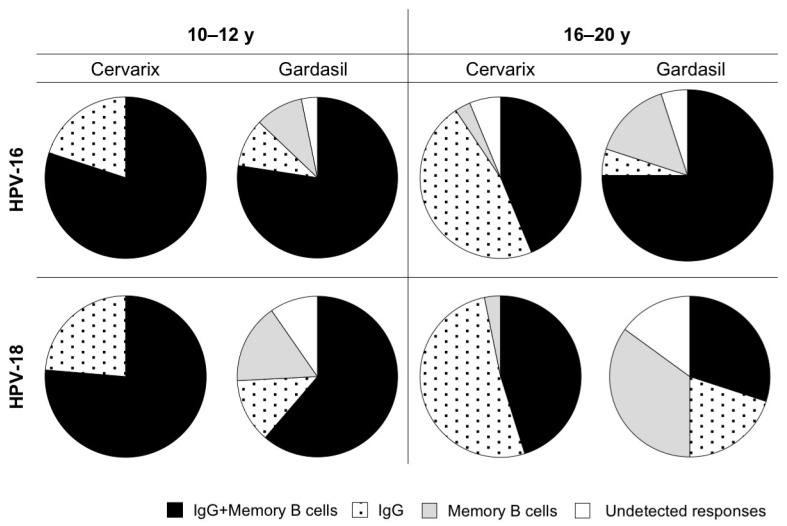
Presence of circulating HPV-16 and HPV-18 specific IgG and memory B cells. Proportion of subjects with both IgG and memory B cells, only IgG, only memory B cells or undetectable responses are shown for each vaccine and age group; for HPV-16 10–12 y, *n* = 40 (Cervarix) and *n* = 31 (Gardasil); for HPV-16 16–20 y, *n* = 32 (Cervarix) and *n* = 20 (Gardasil); for HPV-18 10–12 y, *n* = 38 (Cervarix) and *n* = 31 (Gardasil); for HPV-18 16–20 y, *n* = 31 (Cervarix) and *n* = 20 (Gardasil).

**Figure 6:**
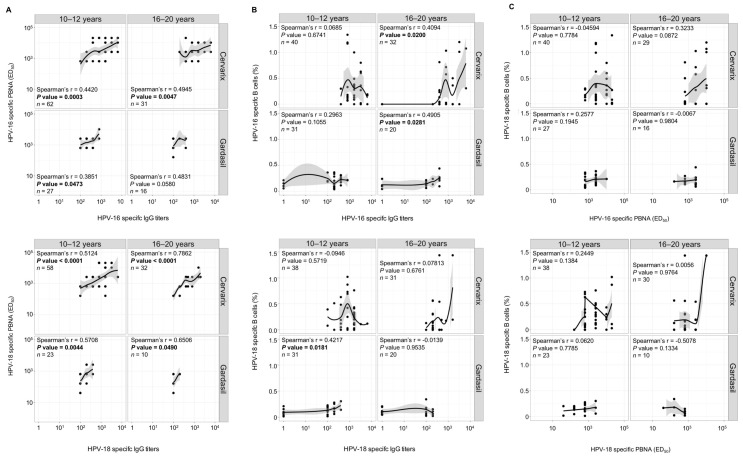
Correlation between HPV-16 and HPV-18 specific IgG, nAbs and memory B cells. Correlation between (**A**) IgG and nAbs, (**B**) IgG and memory B cells and (**C**) nAbs and memory B cells. In each panel, data are stratified by age group (horizontal squares), type of vaccine (top-bottom squares) and HPV subtype (HPV-16, A, B, C, upper panels; HPV-18, A, B, C, bottom panels). Regression lines (dark, full lines) and 95% confidence interval (grey areas) are shown. Spearman’s rank correlation determined statistical significance.

Analyses of circulating antibodies and B cells showed that Cervarix and Gardasil may differently induce these two components of humoral immunity. In particular, the vast majority of Cervarix-vaccinated individuals maintained HPV-specific IgG up to six years after vaccination. However, more than 20% of those aged 10–12 years and more than 50% of those aged 16–20 years had undetectable HPV-specific memory B cells (Figure 5). In addition, very few (3%) Cervarix-vaccinated subjects (16–20 y) had detectable HPV-specific B cells, but not IgG (Figure 5). The latter observation was more frequent in Gardasil-vaccinated subjects, as the percentage of individuals with detectable HPV-specific memory B cells in the absence of circulating antibodies ranged from 10 to 35%, depending on the age group and the HPV subtype (Figure 5). These data suggest that the maintenance of memory B cells and circulating antibodies might be two independent phenomena. We measured the correlation between memory B cell responses, circulating IgG, and nAbs to confirm this hypothesis. While the titers of IgG and nAbs almost always significantly correlated for HPV-16 and HPV-18 for both vaccines and age groups (except for HPV 16 in the Gardasil 16–20 y cohort) (Figure 6A), the same was not true when analyzing the association between IgG titers and memory B lymphocytes. In particular, the HPV-16-specific IgG and B cells levels were only significantly correlated in the 16–20 y age group for both vaccines, while HPV-18-specific IgG and B cells were only significantly correlated in the 10–12 y, Gardasil-vaccinated, subjects (Figure 6B). No significant correlation was instead observed between HPV-specific nAbs and B cells levels, irrespective of the vaccine type, HPV-type and age group (Figure 6C).

The long-term maintenance of circulating IgG is thought to depend on the persistence of long-lived plasma cells (LLPC) or on the differentiation of memory B cells into secreting plasma cells [42,43]. In the latter case, a correlation between antibody titers and memory B cell numbers should be observed. We measured the correlation between HPV-specific IgG and memory B-cells in a cohort of Cervarix vaccinated girls (10–12 y) at short time (1–6 months) after the third vaccine dose to understand if the correlation observed only in one group of vaccinees (Figure 6B) is indicative of a cause-effect relation, or due to the fact that the vaccine may induce both kinds of humoral immunity that may be maintained at a similar extent, yet independently. As shown in Table 1, HPV-16 and HPV-18 specific IgG titers and memory B cells both correlated at short, but not, long time after vaccination. This suggests that the vaccine might induce both PCs and memory B cells in the first phases of the immune response, but these two cell types will then follow different fates. Therefore, circulating antibodies and memory B cells may be independently induced and maintained in a vaccine-specific manner.

## 4. Discussion

The results from this investigator-initiated observational study showed that the humoral responses against HPV vaccine antigens remained detectable at high levels up to six years following the third dose of either Cervarix or Gardasil vaccines. However, the percentages of seropositive subjects and serological parameters (IgG titers, avidity indexes and neutralizing antibody titers) were, overall, significantly higher in both Cervarix age groups when compared to the age-matched Gardasil recipients. The age of the recipients does not influence the differences between vaccines. Notably, we used in-house produced VLPs for testing all samples to assess humoral and memory B cell responses, thus excluding that the differences could be biased by a different antigen source. These data are consistent with previous observations [44,45,46] and are likely dependent on the diverse formulations [47]. Indeed, Cervarix contains AS04 consisting of aluminium hydroxide and MPL, a detoxified derivative of the immunomodulatory cell wall lipopolysaccharide (LPS) molecule of the Gram negative *Salmonella Minnesota* [48] and an agonist of Toll-like receptor (TLR4), which activates the innate immune response [49] and leads to prolonged activation of APCs [50]. Instead, Gardasil only contains the aluminium hydroxyphosphate sulfate adjuvant. Additionally, in agreement with previous observations [45], immune responses against HPV-16 were consistently higher than those against HPV-18 in all Cervarix and Gardasil vaccinated subjects of both age groups. Notably, HPV-18 avidity indexes resulted in being comparable among Cervarix and Gardasil vaccinees implying similar strength of HPV-18 IgG binding independent from the type of vaccine in the long-term after vaccination.

The results also indicated that some of the analyzed immunological parameters and percentage of seropositive subjects in the pre-teen cohorts (10–12 y) tended to be higher than in the young adults cohorts (16–20 y) for both vaccines, which confirmed that the older age of the women enrolled is a parameter that might affect immunogenicity [51,52,53,54]. We also observed high levels of anti-HPV-6 and HPV-11 humoral responses (IgG titers and memory B cells) in subjects who received the Gardasil vaccine approximately six years earlier, which implied a sustainable and strong immunologic response also against the low-risk HPV types elicited by the quadrivalent vaccine and confirming other observations [25]. In addition, long-term cross-neutralizing responses against non-vaccine types, HPV-31 and HPV-45, which lead to approximately 10% of all cervical cancers [55], was determined, and mainly detected in Cervarix-vaccinated women [44,45,46]. As expected, for HPV-31 and -45 the neutralization titers appeared to be lower than those for HPV-16 and HPV-18.

The detection of antigen-specific memory B cells might be indicative of a robust and long- lasting vaccine-induced immune response [56,57], but relatively few studies have examined the proportion and specificity of memory B cells induced by the HPV vaccines [44,58,59,60,61,62]. Strikingly, our results show that, while Cervarix induces significantly higher and more persistent antibody responses when compared to Gardasil, the two vaccines are rather equivalent in inducing memory B-cells; in some cases, the percentage of subjects with vaccine-specific memory B cells was even superior among Gardasil vaccinees (Figure 4A). This results in a proportion of Cervarix vaccinated individuals with circulating antibodies in the absence of detectable memory B cells and, conversely, in a proportion of Gardasil-vaccinated subjects with memory B cells, but undetectable antibodies (Figure 5).

When naïve B cells are activated after the encounter with their cognate antigen, they become either memory B cells or plasma cells (PCs), which can be short- or long-lived PCs (SLPCs and LLPCs) [63]. While the magnitude of early humoral responses is determined by both types of PCs, the long-term maintenance of antibodies is antigen-independent and could last for life [28,63,64], due to the continuous presence of LLPCs. Whether these are the same PCs generated at priming or are rather derived from the differentiation of memory B cells into “new” LLPCs is not clear yet. However, recently, it has been proposed that PCs may survive up to 20 years [65]. In addition, PCs and long-lasting serum antibodies have also been shown to persist in the absence of memory B cells [28,64,66,67]. Together, these evidence suggest that circulating antibodies could be detected, regardless of the presence of memory B cells, as observed in our study for some Cervarix vaccinated women. The independent regulation of circulating memory B cells and antibodies is usually observed in the case of multiple exposure to the antigen (persistent infections or recall vaccine doses), to which IgG titers and memory B cell levels should react differently. We failed to observe in some groups a correlation between circulating IgG titers and memory B cell numbers, consistent with the hypothesis that memory B cells and LLPCs are two independent arms of humoral immunological memory, as also reported for tetanus and diphteria vaccines and for persistent infections, such as EBV and VZV [43,68].

In contrast, it has also been suggested that memory B cells could differentiate (by non-specific stimuli, such as TLRs) into secreting PCs. Consequently, the persistence of IgG would depend on the presence of memory B cells, with a positive correlation between circulating antibody levels and memory B cells [42,43], as in acute infections (Measles, Mumps, and Rubella) and live vaccines (smallpox) [43,69]. We observed a correlation between memory B cells and IgG, for HPV-16, in 16–20 y women for both vaccines, while, for HPV-18, in 10–12 y women only for Gardasil (Figure 6). The lack of a clear pattern suggests that this correlation is an epiphenomenon (circulating IgG and memory B cells share the same stability, even if independently maintained), rather than the conversion of memory B cells into PCs. To support this idea, in the groups where a correlation was observed, we found subjects with IgG, but not memory B cells (for HPV-16 in Cervarix vaccinated, 16–20 y) and memory B cells without IgG (for HPV-16 in Gardasil vaccinated, 16–20 y women and for HPV-18 in Gardasil vaccinated, 10–12 y women). Other studies have, instead, shown a positive correlation between HPV vaccine IgG and memory B cells. This discrepancy could derive by differences in the age of vaccinees, the number of vaccine doses or the time of analysis after vaccination [61,70]. In fact, we have also observed a correlation between HPV-16 and HPV-18 IgG and memory B cells in a cohort of 10–12 y Cervarix vaccinated girls analyzed 1–6 months after the third immunization (Table 1). The correlation appears to be lost when immune responses are analyzed 4–6 years after vaccination in a different cohort of girls vaccinated Cervarix at 10–12 y. Together, these data indicate that HPV vaccination might induce both PCs (including LLPCs) and memory B cells, to a similar extent. However, the two B lymphocyte subsets follow different maintenance kinetics and the correlation is lost over time. The loss of circulating antibodies but not of memory B cells is not uncommon, as also observed for other vaccines, such as against HBV [28,71].

Our observations showed a different pattern between Gardasil and Cervarix. Therefore, it is possible that, during the priming of naïve B cells, Cervarix prompts their differentiation into LLPCs while Gardasil toward memory B cells. The half-life of LLPCs is determined at priming of naïve B cells [72], influencing the antibody persistence [73]. The induction of T follicular helper cells (TFH), the secretion of several cytokines (including IFNγ) and the expression of key transcription factors favor the generation and maturation of antibody secreting cells [74]. The use of MPL in combination with Al(OH)3, in contrast to Al(OH)3 alone, induced higher titers of long-lasting binding and nAbs, while the results of memory B cells were less clear [75]. MPL might also stimulate IFNγ secretion [50,76] and enhance the recruitment of TFH [77]. Notably, Cervarix and Gardasil have shown to induce different subsets of TFH [78] and differently affect PD1 and ICOS expression on TFH [78]. The induction of a precise TFH pattern and the stronger antigenic stimulation and pro-inflammatory cytokine secretion by MPL might account for the preferential differentiation of primed naïve B cells into PCs [79,80], rather than into memory B cells, after Cervarix vaccination, thus also explaining the high antibody titers in the absence of circulating memory B cells.

In conclusion, our results show that the induction and maintenance of circulating antibodies was higher in Cervarix vaccine subjects, and their persistence was memory B cell-independent. However, Gardasil differentiates naïve B cells more toward a memory phenotype. Our results suggest a key role for memory B-cell in protection from HPV when considering that its clinical effectiveness has been shown to persist for at least 10 years [24].

## Figures and Tables

**Table 1:** Correlation between antigen specific IgG titers and memory B cell frequencies.

Antigen	Time after Vaccination	*N* =	Spearman’s r; *p* Value	Slope (95% C.I.)	R^2^
HPV-16	<6 months	53	0.4092; 0.0023	4892 (496; 9287)	0.08932
	>2 years	40	0.06859; 0.6741	75 (−106; 257)	0.01839
HPV-18	<6 months	53	0.5310; <0.0001	3829 (1684; 5975)	0.2014
	>2 years	38	−0.09464; 0.5719	−97 (−407; 213)	0.01109

## Supplementary Materials

The following are available online at https://www.mdpi.com/2076-393X/8/1/26/s1, Figure S1: B cell Elispot protocol optimization, Figure S2: HPV-31 and HPV-45 cross-nAbs, Figure S3: HPV-6 and HPV-11 specific IgG and memory B cells in Gardasil vaccinated subjects.
