# Supplementary material for: HPV-Specific Systemic Antibody Responses and Memory B Cells are Independently Maintained up to 6 Years and in a Vaccine-Specific Manner Following Immunization with Cervarix and Gardasil in Adolescent and Young Adult Women in Vaccination Programs in Italy
Source: Vaccines (Basel). 2020 Jan 14;8(1):26. doi: 10.3390/vaccines8010026 (PMC7175219; doi:10.3390/vaccines8010026)
Supplement: Supplementary file 1 [file vaccines-08-00026-s001.pdf]

Supplementary Materials:

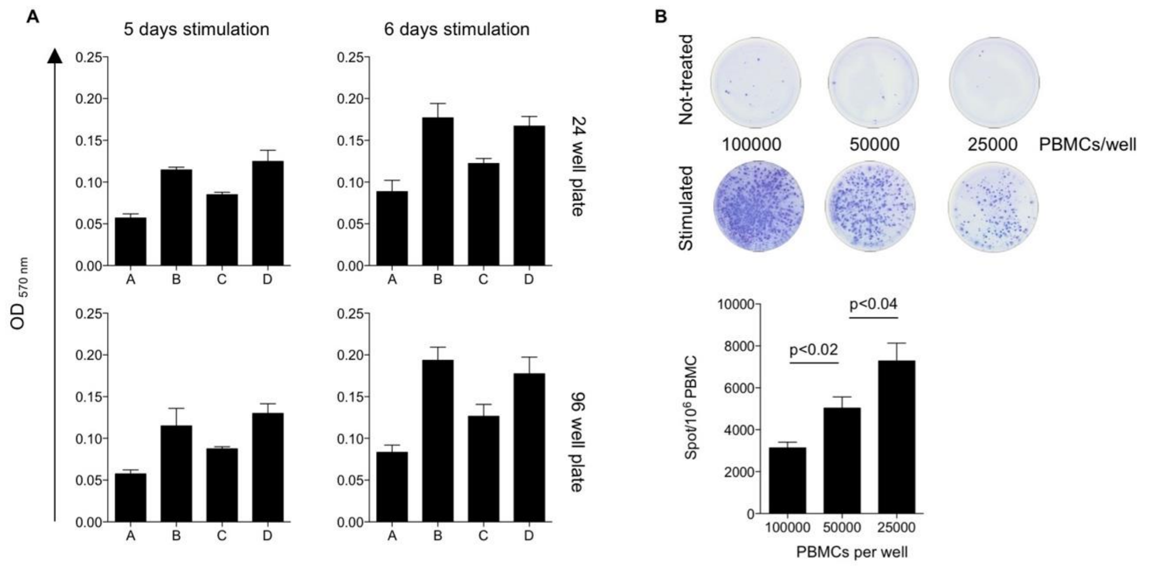

Figure S1

Figure S1. B cell Elispot protocol optimization. (**A**) To set-up the B cell Elispot protocol, we first determined the best conditions in order to differentiate circulating memory B cells into secreting plasma cells. In the first set of experiments, three parameters were evaluated: i) the concentration of stimuli, ii) the well size (24- or 96-well plate) and iii) the optimal duration of the test (5 or 6 days). The polyclonal stimuli defined B, C and D contain an oligonucleotide (ODN 2006), *Staphylococcus aureus* Cowan I and Pokeweed. The concentrations of the different stimuli change from B to D: B contains ODN 2006 (3 µg/ml), *S. aureus* Cowan I (1:10000) and Pokeweed (0.1 µg/ml). C contains ODN 2006 (6 µg/ml), *S. aureus* Cowan I (1:10000) and Pokeweed (1 µg/ml); D contains ODN 2006 (2.5 µg/ml), *S. aureus* Cowan I (1:5000) and Pokeweed (0.06 µg/ml). A constitutes the non-stimulated control. B cell activation was measured by MTT assay. Data are presented as mean + SD from 14 donors (500000 cells/ml). In respect to stimuli concentration, the condition B was chosen as performing like D but requiring a lower amount of reagents. No difference was observed with respect to the well size (96- vs 24-well plates) and length of incubation (5 vs 6 days). We chose to perform all experiments for 5 days in 24-well plates, as higher numbers of cells can be seeded, thus minimizing errors. (**B**) The best number of cells was then determined for B cell Elispot after seeding 100000, 50000 or 25000 cells per well. Spots were too crowded to be counted in wells with 100000 or 50000 cells (top panel), while spots were countable in wells seeded with 25000 cells (bottom panel). We therefore chose this concentration of PBMCs. Data are presented as mean + SD from 14 donors.

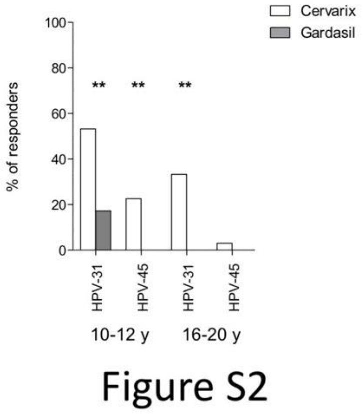

Figure S2. HPV-31 and HPV-45 cross-nAbs. (**A**) Frequency of study subjects with detectable HPV-31 and HPV-45 specific nAbs; for HPV-31 10-12 y, n=62 (Cervarix) and n=29 (Gardasil); for HPV-31 16-20 y, n=33 (Cervarix) and n=20 (Gardasil); for HPV-45 10-12 y, n=62 (Cervarix) and n=29 (Gardasil); for HPV-45 16-20 y, n=33 (Cervarix) and n=20 (Gardasil). Statistical comparisons were made using Fisher's exact probability test.

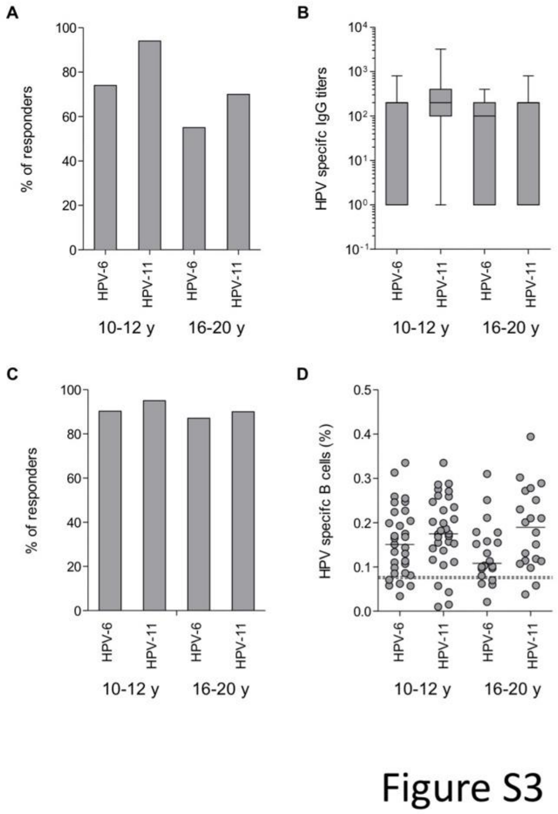

Figure S3. HPV-6 and HPV-11 specific IgG and memory B cells in Gardasil vaccinated subjects. (**A**) Frequency of study subjects with detectable binding HPV-6 and HPV-11 specific IgG. (**B**) HPV-6 and HPV-11 specific IgG titers measured by ELISA shown as box and whiskers plots. (**C**) Frequency of study subjects with detectable HPV-6 and HPV-11 specific memory B cells. (**D**) HPV-6 and HPV-11 specific memory B cells measured by B cell Elispot assay. Each dot represents a single donor, solid lines are the medians and dashed lines are the cut-off values to determine the B cell Elispot positivity. (A-D) n=31 (10-12 y) and n=20 (16-20 y) for both HPV types.
